# Health equity in sickle cell disease: overcoming barriers to care in marginalized communities

**DOI:** 10.1097/MS9.0000000000004195

**Published:** 2025-10-28

**Authors:** Emmanuel Ifeanyi Obeagu, Akash John

**Affiliations:** a Department of Biomedical and Laboratory Science, Africa University, Mutare, Zimbabwe; b Department of Allied Health Sciences, The University of Chenab, Gujrat, Pakistan; c Department of Allied Health Sciences, Lahore Medical Research Center, Lahore, Pakistan

**Keywords:** access to care, healthcare disparities, health equity, marginalized populations, sickle cell disease

## Abstract

Sickle cell disease (SCD) is one of the most prevalent inherited blood disorders globally, disproportionately affecting populations of African, Mediterranean, Middle Eastern, and South Asian ancestry. Despite advances in newborn screening, disease-modifying therapies, and curative interventions, profound disparities persist in diagnosis, treatment access, and survival outcomes. This narrative review examines the structural, socioeconomic, and healthcare barriers to equitable SCD care in marginalized communities and synthesizes policy-relevant strategies to overcome these obstacles. A structured narrative review was conducted by searching PubMed, Scopus, Web of Science, CINAHL, and Google Scholar for literature published between 2010 and 2025. Peer-reviewed articles, policy briefs, and global health reports addressing SCD and health equity were included, with thematic synthesis used to identify key barriers and interventions. Evidence reveals that poverty, inadequate health infrastructure, systemic racism, stigma, and fragmented care systems remain major drivers of inequity. Limited access to newborn screening, hydroxyurea, transfusion programs, and curative therapies perpetuates preventable morbidity and mortality, particularly in sub-Saharan Africa and underserved diaspora communities. Promising interventions include universal newborn screening, community-based education, task-sharing with trained health workers, equitable research funding, and integration of telemedicine to improve specialist access. Health equity in SCD requires more than biomedical innovation. Sustainable progress depends on dismantling structural barriers, strengthening healthcare systems, and implementing policies that prioritize social determinants of health. Coordinated global and local action can transform SCD care from a paradigm of persistent disparity into a model of equitable, patient-centered outcomes.

## Introduction

Sickle cell disease (SCD) is a genetically inherited blood disorder that affects millions of people worldwide, with the highest burden in sub-Saharan Africa, India, the Middle East, and among individuals of African descent in the America and Europe^[1]^. Characterized by the production of abnormal hemoglobin S, SCD leads to chronic hemolytic anemia, recurrent pain crises, organ damage, and early mortality^[2]^. While medical advances have improved outcomes in some high-income settings, significant disparities remain particularly in marginalized communities where resources are limited, and health systems are often underfunded or structurally inequitable^[3]^. Despite its prevalence, SCD continues to be overlooked in global health agendas and national healthcare priorities. Diseases with lower global incidence often receive more funding and attention, reflecting deeper systemic inequities in how healthcare is prioritized and resourced^[4,5]^. In many regions, SCD is still associated with high rates of morbidity and mortality, particularly among children who lack access to early diagnosis, preventive care, or life-saving therapies, such as hydroxyurea, vaccines, and penicillin prophylaxis^[6]^. The avoidable suffering of people with SCD is often shaped less by the disease itself and more by the social and economic conditions in which they live^[7]^.

Health equity is the principle that everyone should have a fair and just opportunity to attain their highest level of health^[8]^. In practice, achieving equity in the context of SCD means addressing the various social determinants of health – such as poverty, education, housing, discrimination, and geographic isolation – that limit access to quality care^[9]^. For many patients with SCD, particularly those in racially marginalized or economically disadvantaged populations, these determinants shape every aspect of their health journey – from timely diagnosis to pain management and life expectancy^[10]^. Marginalized communities often face a complex web of barriers to SCD care. These include limited access to trained hematologists, inadequate healthcare infrastructure, high out-of-pocket expenses, and the geographic concentration of services in urban centers^[11]^. Even in high-income countries, individuals with SCD – especially those from minority backgrounds – frequently encounter healthcare systems that are fragmented, culturally insensitive, or poorly equipped to manage chronic genetic disorders. This contributes to delays in care, suboptimal treatment, and increased risk of complications^[12]^.

Moreover, implicit bias and systemic racism continue to influence clinical decision-making, pain assessment, and treatment allocation^[13]^. Studies have consistently shown that patients with SCD, particularly Black individuals, are more likely to have their pain underestimated, undertreated, or dismissed altogether^[14,15]^. This not only undermines trust in the healthcare system but also results in avoidable complications and repeated emergency department visits. Stigma, both within the medical community and society at large, further marginalizes individuals with SCD and impedes their access to consistent, compassionate care^[16]^. Cultural misconceptions about SCD, particularly in African and South Asian societies, can also hinder care-seeking behaviors and exacerbate psychological distress^[17]^. In some contexts, SCD is misattributed to curses, moral failings, or divine punishment, leading to social ostracism and family conflict^[18]^. Lack of awareness and low health literacy among both patients and providers compound the challenges, contributing to poor adherence to treatment regimens and minimal utilization of preventive services^[19]^.

While biomedical advances hold great promise, achieving equitable outcomes for individuals with SCD requires more than clinical innovation. Addressing the social determinants of health – poverty, education, housing, and discrimination – is essential for closing the survival gap and ensuring that care is accessible, affordable, and culturally responsive. This narrative review critically examines the intersection of health equity and SCD, highlighting the key barriers to care in marginalized communities and synthesizing evidence-based strategies to overcome these obstacles. The objective is to provide policymakers, healthcare providers, and advocates with a comprehensive understanding of the structural drivers of disparity and to outline actionable approaches for promoting equitable, sustainable SCD care worldwide.

## Aim

To explore the health equity challenges facing individuals with SCD in marginalized communities, highlight the social and systemic barriers to care, and propose public health strategies to improve access, outcomes, and equity in care delivery.

## Methods

This review followed a narrative synthesis approach designed to capture the breadth of published evidence on health equity and SCD, with particular attention to barriers affecting marginalized populations. Although not a systematic review, the process incorporated structured elements to enhance transparency and reproducibility.

### Literature search strategy

A comprehensive literature search was conducted between **January and August 2025** using major electronic databases including **PubMed/MEDLINE, Scopus, Web of Science, CINAHL, and Google Scholar**. Search terms combined key concepts related to SCD and health equity using Boolean operators (e.g., *“sickle cell disease”* OR *“sickle cell anemia”* AND *“health equity”* OR *“health disparities”* OR *“marginalized communities”* AND *“access to care”* OR *“barriers to care”*). Reference lists of relevant articles and key policy reports (WHO, Centers for Disease Control and Preventio, African Union) were also hand-searched to identify additional sources.

**HIGHLIGHTS**

- Limited access to comprehensive care contributes to poor outcomes in marginalized populations.
- Implicit bias affects diagnosis, pain management, and treatment.
- Geographic disparities hinder healthcare access.
- Socioeconomic challenges reduce treatment adherence.
- Community engagement and policy reforms are crucial for equity.

### Eligibility criteria

Studies were included if they:

1. Focused on SCD and examined issues of health equity, access to care, or social determinants of health;
2. Reported on marginalized or high-risk populations (e.g., African, Afro-Caribbean, Middle Eastern, Indian, or diaspora communities);
3. Were published in **English** between **2010 and 2025** to capture contemporary evidence and policy contexts.

Eligible publications included peer-reviewed original research (quantitative, qualitative, or mixed-methods), systematic or narrative reviews, policy briefs, and key global health reports. Commentaries without substantive data and non-English sources were excluded.

### Data extraction and synthesis

Two authors independently screened titles and abstracts for relevance, followed by full-text review. Key information extracted included:

- Study setting and population
- Type of barrier(s) described (e.g., socioeconomic, healthcare system, cultural)
- Intervention strategies or policy recommendations
- Key outcomes (e.g., access to screening, treatment adherence, mortality)

Disagreements were resolved by consensus. Findings were synthesized thematically to identify recurring patterns of structural inequity and promising strategies for improving health equity in SCD care.

### Quality considerations

Given the narrative nature of this review, formal risk-of-bias tools were not applied. However, priority was given to peer-reviewed studies and reputable policy sources. Evidence strength was discussed where appropriate to contextualize conclusions.

### Understanding health equity in SCD

Health equity is defined as the principle and goal of ensuring that all individuals have a fair and just opportunity to attain their highest level of health^[20]^. It requires removing obstacles to health such as poverty, discrimination, and their consequences, including lack of access to good jobs with fair pay, quality education and housing, safe environments, and healthcare^[21]^. In the context of SCD, health equity involves addressing the uneven burden of disease and the disparities in access to diagnosis, treatment, and supportive care that exist between different populations^[1]^. SCD predominantly affects racial and ethnic minority groups, particularly individuals of African, Middle Eastern, and South Asian ancestry, many of whom experience social and economic marginalization. This disproportionate impact reflects broader systemic inequities in health and society^[22]^. For patients with SCD, health equity means not only the availability of clinical interventions but also the assurance that social determinants – such as education, income, housing, and social support – are addressed to optimize health outcomes^[23]^.

Achieving health equity in SCD requires recognizing that biological factors alone do not determine health outcomes^[9]^. The interaction between the disease and social context is crucial. Social determinants of health profoundly influence patients’ ability to access quality care, adhere to treatment, and maintain a good quality of life. For example, poverty may limit access to transportation for clinic visits, while low health literacy may affect understanding of disease management^[23]^. Additionally, experiences of racial discrimination and stigma can discourage patients from seeking care or trusting healthcare providers^[24]^. Institutional and structural barriers also contribute to inequities in SCD care. Healthcare systems may lack the resources, trained personnel, and culturally competent services needed to adequately support patients with chronic genetic conditions like SCD^[25]^. Furthermore, implicit biases among healthcare providers can lead to under treatment of pain and insufficient psychosocial support. These systemic issues result in worse health outcomes for marginalized patients and contribute to the persistent health disparities seen in SCD populations^[26]^. Health equity in SCD is not only a clinical or medical challenge but also a social justice imperative. It requires multisectoral approaches that extend beyond the healthcare system to include policy reforms, community engagement, education, and socioeconomic development. Only by addressing both the medical and social dimensions of SCD can equitable and sustainable improvements in health outcomes be achieved^[27]^.

### Barriers to care in marginalized communities

Individuals living with SCD in marginalized communities face a complex array of barriers that hinder access to timely, effective, and comprehensive care. These barriers span economic, geographic, social, and systemic dimensions, collectively contributing to poorer health outcomes and perpetuating health disparities (Table 1).

**Table 1:** Key structural, socioeconomic, and healthcare barriers to equitable SCD Care in Marginalized Communities

Category	Description	Impact on SCD care
Limited access to specialized services	Scarcity of hematologists and specialized clinics, especially in rural or underserved areas.	Delayed diagnosis, lack of preventive care, reliance on emergency services.
Financial constraints	High out-of-pocket costs for medications, hospital visits, and indirect expenses.	Forgone or delayed treatment, increased morbidity and mortality.
Stigma and discrimination	Negative attitudes, racial bias, and misconceptions about SCD leading to undertreatment and mistrust.	Reduced patient trust, inadequate pain management, social isolation.
Low health literacy and cultural barriers	Poor understanding of disease management, language barriers, and cultural beliefs about illness.	Poor adherence to treatment, missed appointments, delayed care.
Fragmented healthcare systems	Lack of integration between primary, specialty, and community care; insufficient provider training.	Discontinuity of care, inconsistent treatment quality.

#### Access and availability of specialized services

One of the primary barriers is the limited availability of specialized healthcare services for SCD, particularly in rural and underserved urban areas^[10]^. Hematologists and specialized clinics that provide comprehensive SCD management – including pain control, prevention of complications, and psychosocial support – are often concentrated in tertiary hospitals located in major cities^[28]^. Patients in marginalized communities may lack transportation or financial means to travel long distances for care, resulting in reliance on emergency services during acute crises rather than receiving preventive, continuous care. This fragmentation undermines disease management and increases hospitalizations and mortality^[29]^.

#### Financial constraints and insurance challenges

Financial barriers represent a significant obstacle to care. The high costs of medications such as hydroxyurea, routine laboratory monitoring, hospitalization, and supportive therapies can be prohibitive for patients without adequate insurance coverage or social safety nets^[30]^. In many low- and middle-income countries, out-of-pocket expenditures dominate healthcare financing, pushing families further into poverty^[31]^. Even in countries with universal health coverage, indirect costs such as transportation, lost income due to illness or caregiving, and childcare can limit access to care. These economic pressures often lead to delayed or forgone treatment, exacerbating disease severity^[32]^.

#### Stigma, discrimination, and implicit bias

Stigma associated with SCD, both within healthcare settings and in broader society, significantly impairs care access and quality. Patients often encounter skepticism or disbelief about the severity of their pain episodes, leading to undertreatment and mistrust of healthcare providers^[33]^. Implicit racial bias further compounds this issue, with studies documenting disparities in pain management and referral patterns for patients of color^[25]^. Beyond the clinical setting, societal misconceptions about SCD contribute to social isolation, discrimination in employment and education, and reluctance to disclose the condition, all of which impact mental health and engagement with care^[24]^.

#### Low health literacy and cultural barriers

Limited health literacy in marginalized populations poses another critical barrier. Understanding the complex nature of SCD, its complications, and the importance of adherence to treatment regimens can be challenging without tailored education and communication strategies^[19]^. Language differences, cultural beliefs about illness, and mistrust of the medical system may further inhibit effective patient–provider interactions. This gap often results in poor adherence, missed appointments, and underutilization of preventive services^[34,35]^.

#### Fragmented and inadequate health systems

Healthcare systems serving marginalized populations frequently suffer from fragmentation and resource limitations^[36]^. A lack of integration between primary care, specialty services, and community-based supports leads to discontinuity in care. Inadequate training of healthcare providers on SCD management, particularly in general practice and emergency settings, results in variable care quality^[37]^. The absence of standardized protocols, registries, and data collection also impedes coordinated care and policy planning^[38]^.

#### Strategies for promoting health equity

Promoting health equity in SCD requires a multifaceted approach that addresses the systemic, social, and clinical barriers faced by marginalized communities^[16]^. Effective strategies must combine improvements in healthcare delivery, policy reform, community engagement, and education to create sustainable change and ensure all individuals with SCD receive comprehensive, culturally sensitive care^[27]^.

#### Strengthening community-based care models

Decentralizing SCD care through community-based clinics and mobile health services can increase access for patients in remote or underserved areas^[39]^. Community health workers trained in SCD management play a crucial role in providing education, facilitating follow-up care, and supporting medication adherence^[40]^. Such models empower patients by bringing care closer to their homes, reducing transportation barriers and promoting continuity of care. Integrating psychosocial support and peer networks within these models can also help reduce stigma and improve quality of life^[41]^.

#### Expanding newborn screening and early intervention programs

Universal newborn screening for SCD is critical for early diagnosis and timely initiation of life-saving interventions, including prophylactic antibiotics, vaccinations, and parental education^[42]^. Early identification enables healthcare providers to monitor patients proactively and implement preventive strategies that reduce morbidity and mortality^[43]^. Scaling up newborn screening programs in resource-limited settings requires government commitment, funding, and infrastructure development, but has proven cost-effective and impactful in improving outcomes^[44]^.

#### Policy reform and advocacy

Advocacy efforts must prioritize the inclusion of SCD in national health policies and universal health coverage schemes. Ensuring that essential medications like hydroxyurea, pain management therapies, and diagnostic services are affordable and widely available is a key step toward equity^[36]^. Governments and stakeholders should also invest in training healthcare professionals and establishing centers of excellence. Advocacy by patient organizations and civil society raises public awareness and influences policymakers to allocate resources and address systemic inequities^[16]^.

#### Enhancing cultural competence and provider training

Healthcare providers need ongoing education to improve their understanding of SCD and to mitigate implicit biases that contribute to disparities in care^[45]^. Cultural competence training fosters respectful, empathetic communication, and strengthens the patient–provider relationship. Equipping providers in primary care and emergency settings with standardized protocols for pain management and SCD complications improves care quality and reduces variability^[46]^.

#### Addressing social determinants of health

Health equity in SCD extends beyond clinical care to include social support systems that address housing, education, employment, and nutrition^[47]^. Multisectoral collaboration between health, social services, education, and community organizations is essential to tackle the root causes of health disparities. Programs that enhance health literacy, reduce stigma, and provide psychosocial counseling empower patients and families to actively participate in disease management^[48]^.

#### Research, data collection, and surveillance

**Figure 1.**
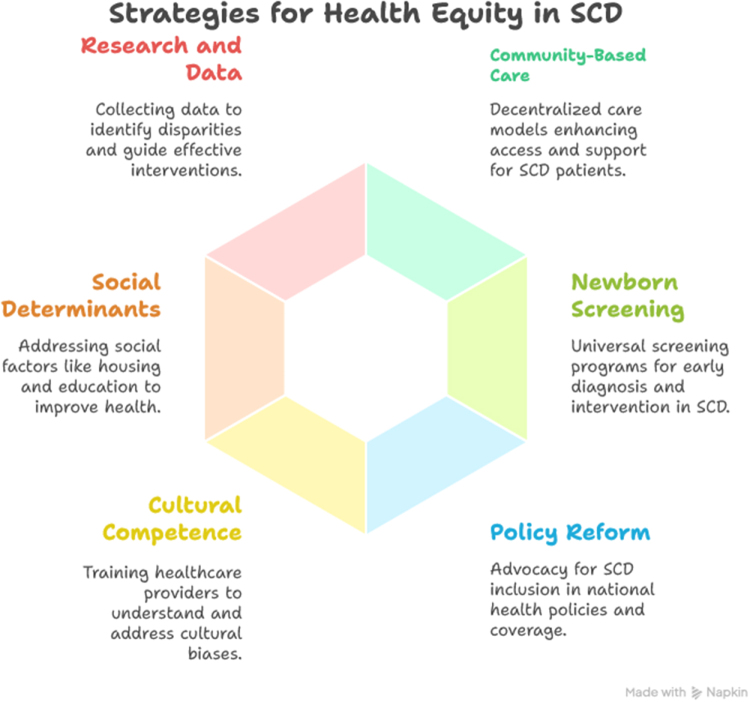
Framework for advancing health equity in SCD: public health strategies across the continuum of care.

Improved data collection and research on SCD within marginalized populations are vital for identifying disparities, monitoring interventions, and guiding policy^[49]^. Establishing patient registries and conducting community-based participatory research ensures that programs are responsive to patient needs and contextually appropriate^[50,51]^. Data disaggregation by race, ethnicity, and socioeconomic status can illuminate hidden gaps and promote targeted strategies^[52]^ (Fig. 1).

## Conclusion

Health equity in SCD remains an urgent global challenge despite significant advances in clinical management. The persistence of structural barriers – such as poverty, inadequate health infrastructure, geographic inequities, and systemic racism – continues to limit access to timely diagnosis, effective therapies, and comprehensive care for marginalized populations. Our review highlights that biomedical innovation alone is insufficient to close the survival gap. Meaningful progress demands a multi-level approach that integrates universal newborn screening, affordable access to disease-modifying and curative treatments, culturally sensitive patient education, and coordinated policy reforms aimed at strengthening health systems.

Community engagement, equitable research funding, and the elimination of implicit bias within healthcare delivery are equally critical to ensure that no individual is disadvantaged by the circumstances of birth or residence. By prioritizing social determinants of health, fostering inclusive public health policies, and scaling proven interventions across resource-limited settings, stakeholders can transform SCD from a disease marked by disparity into a model of equitable, sustainable care. Achieving this vision will require sustained political commitment, cross-sector collaboration, and the active involvement of patients and advocates to ensure that equity becomes a measurable outcome, not merely an aspiration.

## Data Availability

Not applicable as this a narrative review.

## Research registration unique identifying number (UIN)

Not applicable as this a narrative review.
